# Incidence of haematological malignancies in Kosovo—A post "uranium war" concern

**DOI:** 10.1371/journal.pone.0232063

**Published:** 2020-05-04

**Authors:** Hatixhe Latifi-Pupovci, Miranda Selmonaj, Blerina Ahmetaj-Shala, Mimoza Dushi, Violeta Grajqevci

**Affiliations:** 1 Department of Physiology and Immunology, Faculty of Medicine, University of Prishtina, Prishtina, Kosovo; 2 National Heart and Lung Institute, Imperial College, London, United Kingdom; 3 Department of Geography, Faculty of Natural Sciences, University of Prishtina, Prishtina, Kosovo; 4 Department of Paediatrics, Faculty of Medicine, University of Prishtina, Prishtina, Kosovo; European Institute of Oncology, ITALY

## Abstract

**Background:**

During the Kosovo War (1998–99) approximately 31,000 rounds with Depleted Uranium (DU) were fired on 85 targets in Kosovo. The number of haematological malignancies (HM) increased after the war and the concern was the use of DU during the war. The aim of this study was to analyse the incidence rates of HM in Kosovo throughout a 20-year that includes pre- and post- war period (1995–2015); and to examine if there is any association between the use of DU rounds and incidence rates of HM in different regions of Kosovo.

**Methods:**

In this retrospective register-based study, 1,798 new patients diagnosed with leukaemia, Hodgkin lymphoma, non-Hodgkin lymphoma and Multiple myeloma were analysed over a 20 year period. Incidence rates were calculated focusing on specific time periods, regions and age-groups. In addition, the correlation between the use of DU in different regions and their incidence of HM was analysed.

**Results:**

The average annual crude rate of all HM in Kosovo was 5.02 cases per 100,000 persons. Incidence rates of HM in first post-war period (2000–2003) increased by 0.37 cases/100,000 persons (9.51%) compared to the pre-war period (1995–1998) whereas in the last post-war period (2012–2015), incidence of HM increased by 3.19/100,000 persons (82%). Gjakova and Peja, the first and third most exposed regions to DU ordnance ranked first and second in difference in HM. Prishtina, Gjilan and Ferizaj, regions with the least number of rounds/km2, were characterized by a decline of incidence rates.

**Conclusions:**

After the war, the increase in incidence rate of HM was higher in two regions with most DU rounds/km^2^ expended Despite these findings, this study warrants further investigation and does not lead us to a conclusive finding on the existence of a causal relationship between the use of DU during the war and the rise in incidence of HM in Kosovo.

## Background

Depleted uranium (DU) is a by-product created during a uranium enrichment process, and is extensively used for uranium ammunition production [1]. DU ammunition was first used during wars in the Gulf (1991) followed by Bosnia (1994), Kosovo (1999) and Iraq [2]. During the Kosovo War a total of 112 air strikes with DU ammunition were carried out on 96 different targets, of which 85 in Kosovo, 10 in Serbia and 1 in Montenegro [3]. NATO reported that over 31,000 rounds [4] containing at least 10 tons of DU were fired in Kosovo [5]. After the war, a number of studies on DU contamination of the environment (soil, lichen, mushroom, bark etc.) in DU affected areas were published [6–14], with one of them reporting that Kosovo was the most heavily afflicted region by DU pollution in the Balkans [15].

DU has toxicological and radiological effect which, however is lower than that of natural uranium. DU can enter the body by inhalation ingestion (food and water) and by embedded fragments or contaminated wounds [16]. DU exposure has been shown to be toxic to many bodily systems, including brain, kidney, liver and heart [17]. Also, it was shown that DU can affect the immune system inducing shift of Th cells toward Th2, raising suspicion that DU may cause cancer [18]. In the last 20 years, several investigations on DU effects on cells have been conducted which found that overexposure or prolonged exposure of DU is leukemogenic, genotoxic, [19–21], oncogenic[22] and damage to chromosomes[23].

The use of depleted uranium in military actions raised concern after reported increases in incidence of leukemia and lymphoma and other cancers among soldiers involved in the Balkan and the Gulf war [24,25]. In a cancer surveillance study in Italian soldiers deployed in Bosnia and Kosovo, increased incidence of Hodgkin’s lymphoma (HL) was reported, although this was considered as a sporadic event, unlikely related to DU exposure[26]. In contrast, a study carried out in three Iraqi provinces exposed to intensive military activities during the 1991 and 2003 wars, found higher uranium serum levels in leukemia patients compared to control group [27]. However, epidemiological studies which compared the risk of all cancers including leukemia between Balkan veterans and general population in Norway, Sweden, Netherland and Denmark, failed to demonstrate a higher incidence among Balkan veterans [28–32].

There have been indications that the numbers of HM cases in Kosovo post-war have increased and the concern was it was due to the use of DU during the war [33]. The purpose of this study was twofold: 1) to analyse the incidence rates of HM in Kosovo throughout a 20-year pre- and post- war period (1995–2015); and 2) to examine if there is any association between the use of DU rounds and incidence rates of HM in different regions of Kosovo.

## Methods

### Patient data sources

In this retrospective register-based study, we identified all HM newly diagnosed patients in two haematology departments (Clinic of Internal Medicine; January 1995–2015 and the Paediatric Clinic; January 2000–2015) at University Clinical Centre of Kosovo (UCCK)—the only healthcare institution in the country that diagnoses and treats malignancies. In some cases patients may have sought treatment in institutions outside Kosovo, bypassing the UCCK. This particularly applies to four municipalities located in the northern part of the country: Mitrovica North, Leposaviq, Zubin Potok and Zveçan. Nevertheless, we estimate that UCCK covered the vast majority of newly diagnosed patients with hematopoietic and lymphoid malignancies; therefore, the data collected are representative for Kosovo. Due to deficiencies in the Kosovo Health Reporting System, patients’ data were collected from the primary sources—patient registries and records from the two abovementioned clinics. Patient data (age, sex, residence and diagnosis) were cross-checked several times for duplication and inaccurate entries and in dozens of cases were verified via phone calls.

In this study we identified 1989 new patients diagnosed with HM over 20 years in UCCK, of them 1,696 adult patients and 293 paediatric patients. Patients diagnosed with leukemia, HL, NHL and Multiple Myeloma (MM) were the subject of the study. As a result, from the total of 1,989 HM we excluded 129 cases of Myelodisplastic syndrome, 23 cases of Myeloproliferative neoplasms, 12 cases of non-Kosovar patients, 22 cases with largely incomplete data, as well as five cases from two municipalities in the northern part of Kosovo, yielding a total of 1,798 patients analysed.

This study was conducted in accordance with the Declaration of Helsinki. Kosovo Legal Framework requires ethical approval and consent to participate only in the case of bio-medical studies on human subjects [34], whereas the use of personal data for research purposes requires no prior consent from the respective subjects provided the data are anonymised before processing[35], which was the case in this study. Authorization for personal data processing was obtained from the Personal Data Protection Office of the UCCK under the number 2397/2.

### Population data sources

Kosovo is a country of 10,908 km^2^ located in South-East Europe (SEE) with approximately 1.74 million residents. The population is among the youngest in Europe with 47.3% under the age of 25, whereas 62% of population lives in rural areas [36]. In order to analyse the data for HM in the period between 1995–2015 we had to establish relevant population data for each year. There were no population censuses carried out in Kosovo between 1981 and 2011, and huge population movements/ migration occurred during those 30 years due to political tensions, escalation of conflict and post-war situation. The 2011 census was carried out according to the European standards and included only resident population. On the other hand, the 1981 census and subsequent official population estimates carried out in 1991 and 2001 also included the Kosovo Diaspora.

Therefore, based on the 2011 census data, as well as on available growth, migration and Diaspora statistics, we estimated resident populations for the period 1995–2010, whereas official annual estimates of the Kosovo Statistical Agency where used for the period 2011–2015 (S1 Table). Population projections by age groups are available for the period 2011–2015 only and are based on the 2011 census. In this study the medium projection variant was used.

An extraordinary feature of the 2011 population census is the fact that, for political reasons, it was boycotted in 4 municipalities located in the north of Kosovo (Mitrovica North, Leposaviq, Zubin Potok and Zveçan). In addition, population from those municipalities does not seek treatment at UCCK, so it was reasonable to exclude that area from our analysis. The 2011 census provides the only accurate data about Kosovo population by municipalities and regions, so the census data was used for the whole period studied (1995–2015) whenever those variables were analysed.

Kosovo is divided into seven regions (Prishtina, Mitrovica, Prizren, Peja, Gjakova, Gjilan and Ferizaj) and 38 municipalities of which 4 were excluded as described above. From the remaining 34 municipalities, 7 were established from existing ones in the period 2008–2012, and for the sake of continuity, those territories and population were treated as part of the parent municipalities. This focused our analysis on 27 remaining municipalities.

### Geographical exposure to DU

After the war, in 2000, NATO released data on strikes with DU ammunition carried out during the Kosovo War (24 March-10 June 1999) [37], including coordinates of the sites and number of rounds used in the majority of strikes [3]. To identify municipalities where DU ordnance was expended and analyse proximity of targeted locations to settlements, we converted NATO coordinates to the DMS format and used Google Maps. From the available data we can conclude that a total of 97 strikes throughout Kosovo were carried out by DU rounds. Four strikes were excluded due to missing or erroneous coordinates which made it impossible to determine the location. From remaining strikes, it is known that in 76 of them, a total of 26,373 DU rounds were expended, whereas data on the number of rounds expended in the remaining 17 strikes are not available. Therefore, for each such strike we have assigned the average number of rounds in the 76 strikes where such data is available—347. This brings us to an estimate of 5,899 rounds expended in 17 strikes, and the total number of 32,272 expended throughout Kosovo. In total, 20 municipalities were affected by air strikes with DU rounds, and for those municipalities the average numbers of rounds per km^2^ were computed (Table A in S2 Table). On the other hand, there were no reported strikes in the 7 remaining municipalities.

### Statistical analysis

Descriptive findings are presented for 1,798 patients diagnosed with leukemia, HL, NHL and MM during the period 1995–2015. Annual incidence crude rates per 100,000 persons/year were calculated for each disease separately, as well as for HM combined. For the sake of accuracy, annual incidence rates at Kosovo level were calculated by using population data estimates for respective years, whereas calculation of annual incidence rates for the 7 Kosovo regions is based on the 2011 census data. ‘Age Specific Incidence Rate’ was calculated only for the period 2011–2015 using population projections [38] based on the 2011 census, because of missing age-specific data for the period 1995–2010. In this study the age distribution of the population is given in five-year age groups extending to 75 years and over. For the same reason (missing age-specific data for the period 1995–2010), ‘Age-Standardised Rates’ (ASR) according to European Standard Population are presented only for the period 2011–2015, for the sake of comparison of incidence rates with different countries.

In order to compare different findings over time, we defined five time periods: 1995–1998, 2000–2003, 2004–2007, 2008–2011, 2012–2015. The year 1999, when the 11-weeks NATO air-campaign effectively ended the war in Kosovo, was excluded from those time periods due to huge displacements of population and considerable disruption in provision of health care services in Kosovo.

To analyse changes in incidence throughout different time periods and regions, we calculated differences of average annual incidence between the pre-war period (1995–1998) and two post-war periods—the first (2000–2003) and the last (2012–2015). (Table D and Table E in S3 Table).

Statistical Analysis was performed by SPSS, version 23 and Microsoft Excel 2010, version 14. Data were plotted on Graphpad Prism, v5.

## Results

### Descriptive characteristics of HM in Kosovo, 1995–2015

The distribution of 1,798 patients diagnosed with leukemia, HL, NHL and MM in Kosovo for the period 1995–2015 is presented in Table 1, along with median age at the time of diagnosis, sex-rate ratio (male rate/female rate), average annual incidence CR/100,000 and annual average ASR only for the period 2011–2015. The most frequent type of HM was leukemia with 49.5%. HM were more frequent in males than in females (sex-rate ratio 1.32) with difference being more evident for HL (sex-rate ratio 1.54). The median age at diagnosis of HM combined was 50 years. The average annual CR of all HM in Kosovo was 5.02 cases per 100,000 persons, whereas the average annual ASR for period 2011–2015 for HM combined was 8.33.

**Table 1:** Characteristics of HM in Kosovo for a 20 year period (1995–2015).

Diagnosis	Frequency N (%)	Median age at diagnosis (years)	Sex-rate ratio (m/f)	Average Annual CR rate per 100 000, 1995–2015			Average annual ASR 2011–2015		
				M	F	T	M	F	T
Leukemia	890 (49.5%)	43 (16–62)	1.30	2.81	2.16	2.49	4.52	2.71	3.57
HL	251 (14.0%)	44 (27–60)	1.54	0.85	0.55	0.70	1.32	0.81	1.06
NHL	468 (26.0%)	54 (41–65)	1.27	1.45	1.15	1.30	2.94	2.23	2.56
MM	189 (10.5%)	61 (51–67)	1.32	0.60	0.45	0.52	1.55	0.76	1.13
Total HM	1,798 (100.0%)	50 (25–64)	1.32	5.71	4.32	5.02	10.33	6.51	8.33

### Age-specific incidence rates of HM

**Fig 1:**
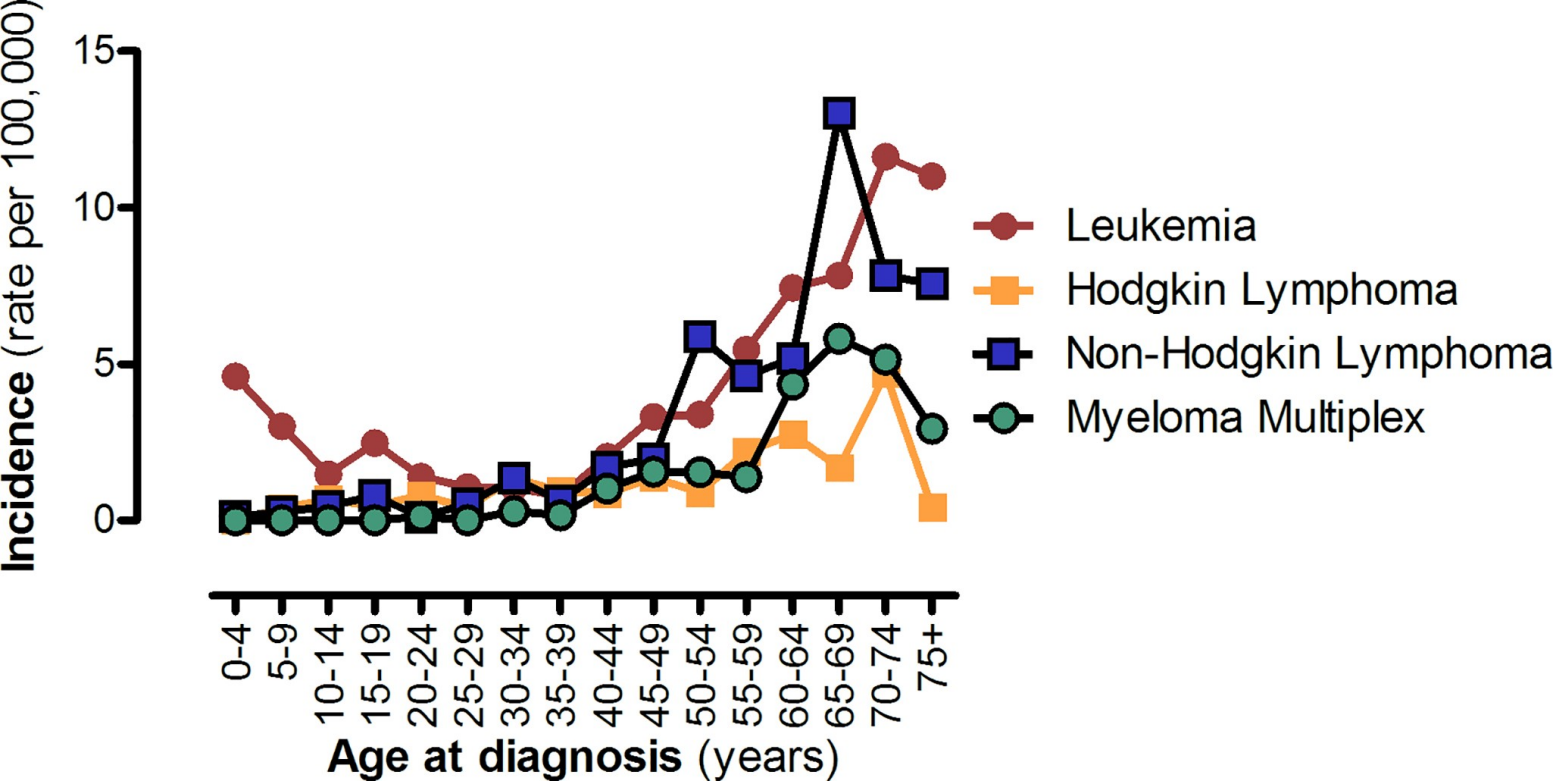
Average annual age-specific rates for HM in Kosovo for the period 2011–2015. Incidence rates of leukemia, Hodgkin’s Lymphoma, Non-Hodgkin’s Lymphoma and Myeloma Multiplex between 2011–2015.

Leukemia was the most common malignancy among children and adults till the age of 30, but the majority of cases occurred in older people (Fig 1). NHL exceeded leukemia at ages 30–34, 50–54 and 65–69. The decline in incidence of NHL and MM occurred at ages 65–69 whereas the decline in incidence of leukemia and HL occurred at ages 70–74.

### Regional variations in incidence of HM during five four-year periods

In the pre-war period, the incidence rates of HM malignancies combined were higher in Prishtina, Mitrovica and Ferizaj with increased prevalence of leukemia (3.23) and HL (0.92) in Prishtina, MM (0.65) in Mitrovica and NHL (1.12) in Ferizaj. In this period the lowest incidence of HM combined was in Gjakova and Peja (Table B in S3 Table). In the last post-war period (2012–2015), Mitrovica was still the region with the highest incidence of HM (9.34), followed by Ferizaj (8.11), Peja (8.06) and Prishtina (7.87) (Table B in S3 Table). Incidences for each malignancy for the same time periods were also calculated (Table B in S3 Table).

**Fig 2:**
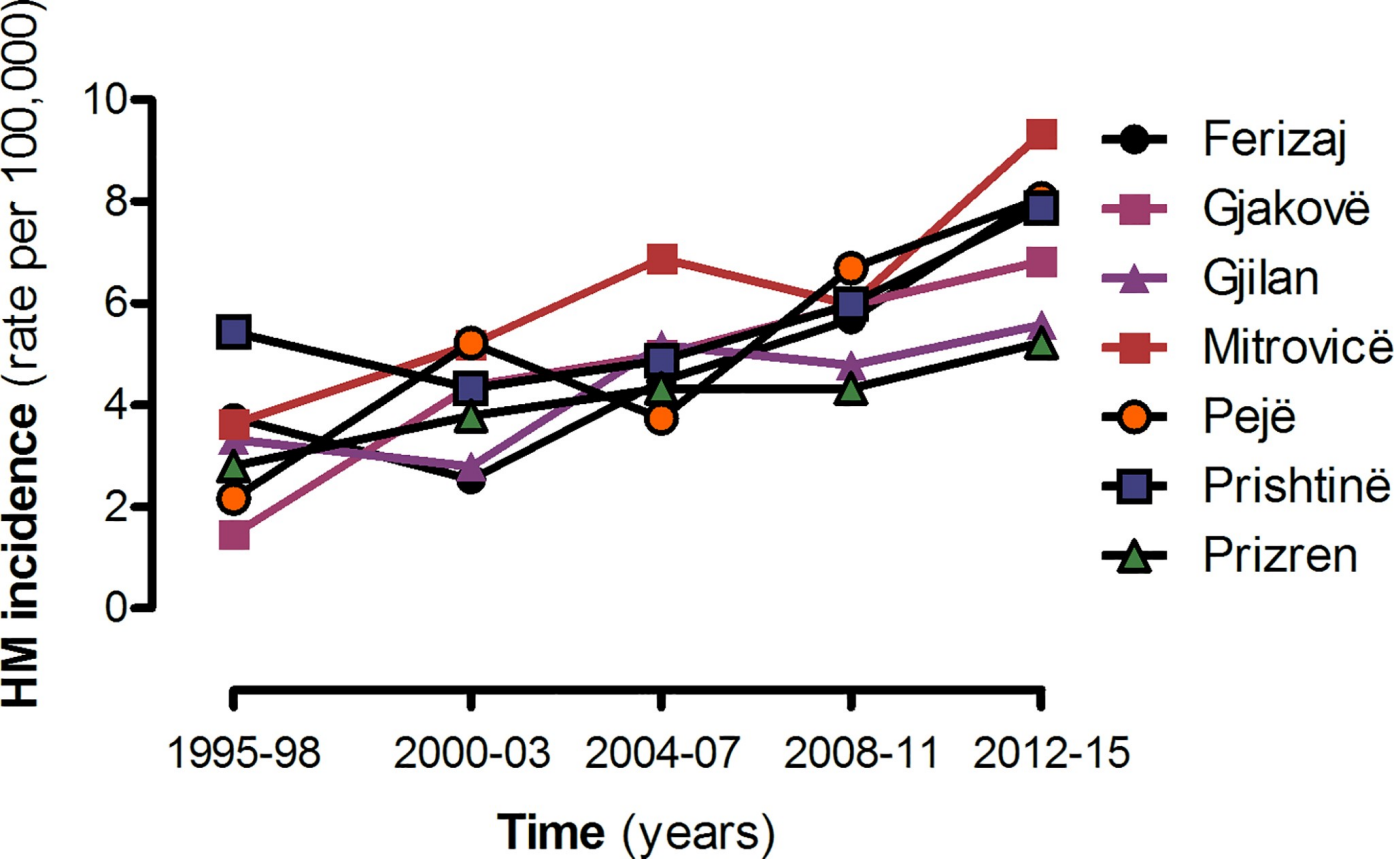
Average annual incidence rates/100 000 of HM combined by regions and time periods, 1995–2015. Incidence rates (per 100,000 people) in 7 different regions (Ferizaj, Gjakova, Gjilan, Mitrovica, Peja, Prishtina and Prizren) of Kosovo were investigated from 1995–2015.

After the war, incidence rates of HM combined increased continuously throughout the five-year time periods. The post-war changes can be summarised as follows; the first post-war period and third post-war period (2000–2003 and 2008–2011) were characterised by sharp increases in leukemia and NHL in the majority of regions of Kosovo, the second post-war period (2004–2007) with increases in HL and MM in the majority of regions, whereas the fourth post-war period (2012–2015) with increases of all malignancies in the majority of regions of Kosovo (Fig 2).

### Difference in incidence of HM between pre-war period and the two post-war periods and DU exposure by regions

Table 2 presents incidence rates by seven Kosovo regions in the pre-war (1995–1998), as well as the first and the last post-war period (2000–2003 and 2012–2015). In the pre-war period the lowest incidence rates of HM combined were in the two regions most exposed to DU ordnance (Gjakova and Peja), whereas the highest incidence rates were encountered in the regions with lower exposure to DU ordnance (Prishtina and Mitrovica). Whereas, in the first post-war period (2000–2003) Gjakova and Peja, the first and the third most exposed regions to DU ordnance show proportionally higher increase in incidence compared to the pre-war period (1995–1998) than it is the case with the regions least exposed to DU ordnance—Prishtina, Gjilan and Ferizaj which were characterized by declining in incidence compared to the pre-war situation. The increase in incidence of HM combined also was shown in Prizren region which ranked second by exposure to DU and Mitrovica which ranked fourth. Similar trends can be observed in increase of incidence rates of Leukemia in the respective regions.

**Table 2:** Regional variations of rounds/km2 and incidence in the pre-war period (1995–1998) and two post-war periods (2000–2003 and 2012–2015).

Region	Rounds/km2	Leukemia			Hodgkin Lymphoma			Non-Hodgkin Lymph.			Myeloma Multiplex			All malignancies		
		1995–1998	2000–2003	2012–2015	1995–1998	2000–2003	2012–2015	1995–1998	2000–2003	2012–2015	1995–1998	2000–2003	2012–2015	1995–1998	2000–2003	2012–2015
Ferizaj	1.58	1.72	1.01	3.55	0.51	0.61	1.62	1.12	0.71	1.93	0.41	0.20	1.01	3.75	2.54	8.11
Gjakova	7.73	0.49	2.80	3.53	0.49	0.12	0.85	0.24	0.97	1.46	0.24	0.49	0.97	1.46	4.38	6.82
Gjilan	0.73	1.46	1.46	2.92	0.40	0.27	0.27	1.06	0.93	1.99	0.40	0.13	0.40	3.32	2.79	5.58
Mitrovica	1.85	1.43	2.98	3.63	0.52	0.39	0.78	1.04	1.56	2.47	0.65	0.26	2.47	3.63	5.19	9.34
Peja	4.78	0.68	1.82	3.74	0.57	1.02	1.13	0.68	2.04	2.50	0.23	0.34	0.68	2.16	5.22	8.06
Prishtina	1.17	3.23	2.07	3.78	0.92	0.67	0.85	0.79	1.22	2.38	0.49	0.37	0.85	5.43	4.33	7.87
Prizren	5.42	1.44	2.17	1.90	0.27	0.45	1.26	0.99	0.99	1.26	0.09	0.18	0.81	2.80	3.79	5.23

Increase in HM continued over the 15 post-war years. This was reflected in big differences seen in incidence rates between the pre-war (1995–1998) and the last post-war period (2012–2015). Throughout the Kosovo region, HM combined increased by 3.19 cases/100,00 persons (82%) (Table 2). When analysed by regions, we found that the largest increase of incidence of HM combined occurred in Gjakova and Peja, with the largest difference seen in leukemia and NHL. Prizren was the region with the largest increased incidence of HL and MM. The smallest increase of incidence of HM combined was found in Prishtina and Gjilan.

## Discussion

To date, this is the first study which analyses incidence of four broad categories of HM—leukemia, NHL, HL and MM in Kosovo for a period of twenty years (1995–2015). In Kosovo the most frequent type of HM was leukemia, which was similar to that seen in most other SEE countries—Albania, Greece, BiH, Macedonia, Serbia and Montenegro, excluding Slovenia and Croatia where the most frequent type of HM was NHL [39].

Similar to other SEE countries and estimates for Albania, Greece, Macedonia and Montenegro[39], incidence of all HM in Kosovo were higher in males than in females. Age of diagnosis of HM varies across different world regions. In western countries HM usually affects older people, whereas in Asia it is diagnosed at younger ages [40–42]. The decline in incidence rates of HM in Kosovo occurred earlier than in other SEE countries, presumably as a result of shorter longevity (life expectancy 71.65 years) [43].

Incidence rates of HM vary widely by geographic location suggesting different etiological factors, and, in general, are higher in more developed countries [39,44]. Compared to other SEE countries [39], Kosovo had the lowest ASR of all HM, except for NHL and MM which were higher only compared to Albania. It is known that higher national income and healthcare budgets are associated with the higher cancer incidences [45]. From that perspective, low incidence rates of HM in Kosovo may be explained by the fact that it is lower-middle-income country [46] with a poor healthcare system and variations in malignancy detection. Whereas, a possible explanation for the low incidence of lymphomas can be the low incidence of HIV infection in this country [47] which is known as a risk factor for lymphomas [48–51].

Although the results of the present study demonstrate that the incidence of all HM in Kosovo was lower than in other countries, in the 20-year period, the incidence rate increased more compared to other countries. Also, in general, in Western countries the overall incidence of HM appears to be rising [52]. In SEE countries some HM showed increase in incidence rates and others decrease in incidence. For example, in Slovenia, the CR of leukemia increased by 28.1% in 2012 compared to annual average CR in period 1995–1998, NHL by 78.2%, MM by 94.5% whereas HL decreased by 19.5%. In Croatia, the annual crude rate of NHL increased by 29.3% in 2012 relative to average annual CR in period 2001–2004, MM by 5.9% whereas leukemia and HL decreased by 8.1% and 11.5% respectively [44]. The larger increase in incidence of HM in Kosovo may partly be due to improvements in diagnostic procedures and the increase in longevity, but also may reflect a true increase in disease occurrence. Due to the fact that Kosovo is the most heavily afflicted region by DU pollution in the Balkans [15], possibility of DU toxicity and radiation effects on inhabitants cannot be ruled out, although it is far from being proven.

Analysis of pre-war incidence rates show that the lowest incidence rates of HM combined were in the two regions most exposed to DU ordnance (Gjakova and Peja), whereas highest incidence rates were encountered in the regions with low exposure to DU ordnance (Prishtina and Mitrovica). Prishtina and Mitrovica are considered to be the most polluted regions in Kosovo, due to the presence of polluting industries which is one of the factors explaining the pre-war difference in incidence of HM between regions. The Mitrovica region is known as one of the most polluted areas in Europe by several toxic heavy elements such as Pb, Zn, As and Cd, as a consequence of a lead processing industry which operated until the year 2000 [53–55]. Also, a study by Di Lella et al. in 2001 showed that levels of uranium analysed in lichens from Mitrovica were higher than in other regions of Kosovo, suggesting contamination of thalli by terrigenous material and not by DU contamination of the environment [55]. On the other hand, in the region of Prishtina there are coal power plants which are the largest point source of air pollution in Europe [56]. In a study by Kittner et al. significant trace metal content—Ni, Cr, Hg, As was found in lignite coal from Obiliq municipality (12 km far from Prishtina) [56]. Knowing that high exposure to methylmercury is associated with leukemia [57,58] and that there were suggestions that Ni would be an etiological factor for leukemia [59], it is possible that higher incidence rates of leukemia in pre-war and post-war period in Prishtina, could be partially attributed to environmental pollution by these heavy metals.

Despite the fact that before and after the war Mitrovica and Prishtina were regions with the highest incidence of HM, the differences in incidence between the pre-war period and the first and last post-war period, were highest in the two least polluted regions of Kosovo, but with most DU ordnance expended per km^2^—in Gjakova and Peja. On the other hand, two regions with the least number of rounds/km2, Prishtina and Gjilan, in the first and last post-war period had a decrease in incidence of HM.

If we take into account that DU can enter the body by ingestion (food and water), increased levels of uranium into the water could pose risk for population. After the war in Kosovo, several research groups analysed uranium water concentration. In a study done in the Region of Gjakova in 2001 by Mellini and Riccobono, uranium levels analysed in 247 wells showed normal values[60]. On the other hand, in a study done in 2014 by Berisha et al. which analysed 951 water samples, mainly in central and eastern part of Kosovo, only 2.6% of samples exceeded the World Health Organization maximum acceptable concentration of 30 μgL/1, and 44.2% of the samples exceeded the 2 μgL/1 German maximum acceptable concentrations recommended for infant food preparations [61]. Whereas immediately after the war levels of uranium in water were within normal values, high contamination of soil samples taken in battlefield areas in southwest Kosovo[6,7,13,62] was found, which could enter the food chain. However, it is not possible to conclusively attribute this findings because we have assessed the exposure to DU at the municipality level and not individual level, and due to the fact that DU is not considered as a human carcinogen[63]. Namely, uranium is not very radioactive (having such a long half-life of billions of years, 238U decays very slowly), and its chemical properties are often such that any inhaled or ingested uranium is excreted rather quickly from the body[64].

Nonetheless, there are several contradictory studies about DU effect on the risk of occurrence of cancers among them leukemia and lymphomas. A small number of studies show the increased risk [25, 26], whereas a huge number of epidemiological studies provide no consistent evidence that uranium causes leukemia or lymphoma such are occupational studies of workers exposed to uranium for many years, studies of cancer mortality and cancer incidence among people who have lived near uranium mills, mines and processing facilities and studies of military participants and peacekeepers [64,65–68].

Whilst this study is the first of its kind in Kosovo, there are several limitations. Firstly, there were no cancer registry data in Kosovo for the period analysed, so data were collected from patient registries and records. Secondly, deficiencies in demographic data required us to resort to estimates of resident populations for the period 1995–2010 which may not always be accurate. Since it was not possible to estimate data by age groups for this period, the crude rates were used instead of age-standardised rates. Thirdly, four Kosovo municipalities constituting around 9% of the territory were excluded from this study due to the fact that populations living in those areas do not seek medical treatment at UCCK.

## Conclusions

To date, this pioneering study of HM incidence analysis in Kosovo showed an increase by 3.19 cases/100,000 persons (82%) between the 20 years period of 1995–2015. The differences of HM incidence between pre and post war periods were higher in two regions with most DU rounds/km^2^ expended. Despite these findings, this does not lead us to conclusive findings on the existence of causal relationship between the use of DU during the war and the rise in incidence of HM in Kosovo. So, further studies need to take place in specific regions of Kosovo to come to conclusive findings.

## Supporting information

S1 Table Estimate of the resident population in Kosovo for the period 1995–2015. (XLSX)

S2 Table Average numbers of DU rounds per km2 by municipality and region. (XLSX)

S3 Table Incidence for hematological malignancies by regions and time periods. Supporting file for Table 2 and Fig 2.
(XLSX)

S4 Table Hematological malignancies in Kosovo 1995–2015. Supporting file for Table 1 and Fig 1.
(XLSX)
